# West Nile and Usutu Virus Infections and Challenges to Blood Safety in the European Union

**DOI:** 10.3201/eid2506.181755

**Published:** 2019-06

**Authors:** Dragoslav Domanović, Celine M. Gossner, Ryanne Lieshout-Krikke, Wolfgang Mayr, Klara Baroti-Toth, Alina Mirella Dobrota, Maria Antonia Escoval, Olaf Henseler, Christof Jungbauer, Giancarlo Liumbruno, Salvador Oyonarte, Constantina Politis, Imad Sandid, Miljana Stojić Vidović, Johanna J. Young, Inês Ushiro-Lumb, Norbert Nowotny

**Affiliations:** European Centre for Disease Prevention and Control, Solna, Sweden (D. Domanović, C.M. Gossner, J.J. Young);; European Blood Alliance, Amsterdam, the Netherlands (R. Lieshout-Krikke);; Austrian Red Cross, Vienna, Austria (W. Mayr, C. Jungbauer);; National Competent Authority for Blood, Budapest, Hungary (K. Baroti-Toth);; National Competent Authority for Blood, Bucharest, Romania [external expert] (A.M. Dobrota);; National Competent Authority for Blood, Lisbon, Portugal (M.A. Escoval);; Paul Ehrlich Institute, Langen, Germany (O. Henseler);; Italian National Blood Centre, National Institute of Health, Rome, Italy (G. Liumbruno);; National Competent Authority for Blood, Madrid, Spain (S. Oyonarte);; Hellenic Center for Disease Control and Prevention (KEELPNO), Athens, Greece (C. Politis);; National Competent Authority for Blood, Paris, France (I. Sandid);; Croatian Institute for Transfusion Medicine, Zagreb, Croatia (M.S. Vidović);; National Health Service Blood and Transplant (NHSBT), London, UK (I. Ushiro-Lumb);; University of Veterinary Medicine, Vienna, Austria (N. Nowotny);; Mohammed Bin Rashid University of Medicine and Health Sciences, Dubai, United Arab Emirates (N. Nowotny)

**Keywords:** West Nile virus, Usutu virus, blood safety, surveillance systems, measures, European Union, viruses

## Abstract

West Nile virus (WNV) and Usutu virus (USUV) circulate in several European Union (EU) countries. The risk of transfusion-transmitted West Nile virus (TT-WNV) has been recognized, and preventive blood safety measures have been implemented. We summarized the applied interventions in the EU countries and assessed the safety of the blood supply by compiling data on WNV positivity among blood donors and on reported TT-WNV cases. The paucity of reported TT-WNV infections and the screening results suggest that blood safety interventions are effective. However, limited circulation of WNV in the EU and presumed underrecognition or underreporting of TT-WNV cases contribute to the present situation. Because of cross-reactivity between genetically related flaviviruses in the automated nucleic acid test systems, USUV-positive blood donations are found during routine WNV screening. The clinical relevance of USUV infection in humans and the risk of USUV to blood safety are unknown.

West Nile virus (WNV) and Usutu virus (USUV) are arthropod-borne flaviviruses that belong to the Japanese encephalitis serocomplex. Their natural life cycle involves ornithophilic mosquitoes (predominantly *Culex* spp.) as vectors and birds as amplifying hosts. Mammals, including horses and humans, may act as accidental hosts. In temperate climate zones, WNV and USUV usually circulate from late spring to mid-autumn, when competent mosquitoes are active.

WNV was detected in Europe in 1958 (*1*). Among its 9 evolutionary lineages (*2*), WNV lineages 1 and 2 are the most notable. Most recent outbreaks of WNV infections in the European Union (EU) are caused by the central European lineage 2 WNV, which was introduced to southeastern Hungary in 2004 (*3*) and has spread to other countries in Europe since 2008 (*4*,*5*). Another (eastern European) lineage 2 WNV was independently introduced to the Volgograd area of Russia at approximately the same time (*6*) and spread from there to Romania (*7*) and Italy (*8*). In addition, lineage 1 WNV strains are still circulating in Europe, partly overlapping with lineage 2 strains (*8*,*9*). Around 80% of WNV infections in humans are asymptomatic (*9*,*10*). Most clinical WNV infections present with mild, influenza-like symptoms, and ≈1% of infected persons, most often the elderly or immunosuppressed, develop West Nile neuroinvasive disease (WNND), which causes variable rates of illness and death (*9*,*10*). 

In humans, transmission of WNV through blood transfusion, organ transplantation, breast-feeding, and intrauterine means has been reported (*11*–*14*). WNV poses a risk to blood safety because an asymptomatic donor may donate infectious blood, which, if transfused, may cause a serious clinical illness in the recipient. To mitigate such risk, EU countries apply measures described in the EU Directives (*15*), professional guidelines (*16*), and the European Centre for Disease Prevention and Control (ECDC) coordinated blood safety preparedness plan (*17*). As part of these actions, blood banks should apply blood safety measures directed to donors residing in or visiting areas affected by WNV (*17*). An area with >1 confirmed case of autochthonous WNV transmission to humans is considered affected (*18*). To support EU countries in applying legislation related to travelers visiting affected areas, the ECDC has developed web-based maps indicating areas at Nomenclature of Territorial Units for Statistics (NUTS) level 3 in the EU where confirmed cases of WNV infections in humans have been reported (*19*).

USUV is a mosquitoborne flavivirus closely related to WNV that was reported in Europe as a cause of death in birds (mainly blackbirds) in and around Vienna, Austria, in 2001 (*20*). It was also retrospectively identified as the etiologic agent of blackbird die-off in the Tuscany region of Italy in 1996 (*21*). Since then, the virus has spread across Europe (*22*,*23*). Clinically manifested cases of USUV infection in humans are rarely detected. In 2003, USUV-specific nucleic acid was identified in the blood of a young man with a rash in a USUV-endemic area around Vienna (*24*). In 2009, two human cases of USUV-related neuroinvasive illness were reported in Italy (*25*,*26*), and in 2013, three other human cases were reported in Croatia (*27*). USUV has also been recently associated with a clinical diagnosis of idiopathic facial paralysis in France (*28*). Transfusion-transmitted USUV infection has not been reported.

We evaluated the safety of the blood supply during WNV outbreaks in the EU by summarizing the preventive strategies applied, the functional use of WNV infection distribution maps by blood banks and responsible blood safety authorities, the occurrence of WNV infections among blood donors, and cases of transfusion-transmitted WNV (TT-WNV) infection. Because USUV circulates or cocirculates with WNV in certain EU countries (*29*) and current virus RNA detection systems show cross-reactivity between these viruses, we also discuss the threat posed by USUV to blood safety.

## Materials and Methods

ECDC organized an expert meeting in Vienna in March 2018. Experts and representatives of the National Competent Authorities for blood and blood components from 11 countries in Europe presented relevant data, which we used in this evaluation. The European Blood Alliance provided data on preventive measures from the other EU member states. We also reviewed the scientific literature on blood donation and transfusion-related WNV and USUV infections and retrieved data on reported cases of WNV infection in the EU population from The European Surveillance System (TESSy) of ECDC.

## Results

According to data reported to TESSy during 2009–2017, a total of 1,757 cases of WNV infection, with an annual range from 30 to 356 cases, were reported in the EU countries (Figure). Most cases (1,695) were reported to be locally acquired; only 62 cases were imported (*30*). We summarized data on WNV infection among blood donors, which were not consistently reported. Detailed descriptions of blood safety measures and frequencies of WNV and USUV-positive blood donations in the EU are provided in the Appendix.

**Figure:**
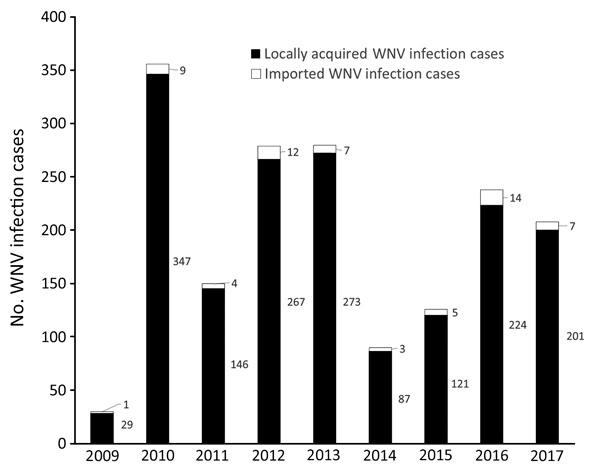
Annual number of locally acquired and imported WNV infection cases in the European Union reported to the European Surveillance System during 2009–2017. WNV, West Nile virus.

### Preventive Strategies

As of August 31, 2018, most EU countries (15/17; 88%) without local WNV transmission to humans apply a 28-day deferral of asymptomatic blood donors after they leave an area with ongoing transmission of WNV to humans. These countries defer donors with a travel history to WNV-affected areas within EU at NUTS 3 level or at a country level in affected non-EU countries such as the United States, Canada, and countries in the tropical zone (delimited by latitudes 23°26′12.5″ in the Northern and Southern Hemispheres). The minimum time spent in an affected area that countries consider as a deferral criterion varies from 1 night to 2 consecutive days. As alternatives to the 28-day deferral, the United Kingdom uses a mini-pool (MP) of 6 samples and Ireland performs individual donation (ID) WNV screening using nucleic acid testing (NAT) of blood donations from traveling donors who have visited a WNV-affected country in the previous 28 days during the mosquito season (May–November). These methods are used to avoid donor shortages resulting from deferral and the cost incurred to maintain an adequate donor base by recruiting new donors. Some blood banks in Germany perform either MP or ID-NAT screening of donations from travelers to affected areas.

As of August 31, 2018, a total of 11 of 28 (39%) EU countries have reported local autochthonous transmission of WNV; 9 (81%) of those countries implemented NAT screening of blood donations (Table 1). For purposes of implementation of local blood safety measures, geographic determination of an area in which a vectorborne disease is present is based on epidemiologic analysis and risk assessment (*18*).

**Table 1:** Occurrence of locally transmitted or imported cases of West Nile virus infection and blood safety measures by country in the European Union as of August 31, 2018*

Country	Affected areas with local transmission cases (measure)	Nonaffected areas with imported cases (measure)
Austria	+ (MP-NAT)	0 (D/MP-NAT†)
Belgium	0	0 (D)
Bulgaria	+ (D)	0 (D)
Croatia	+ (ID-NAT‡)	0 (D)
Cyprus	0	0 (D)
Czech Republic	0	0 (D)
Denmark	0	0 (D)
Estonia	0	0 (D)
Finland	0	0 (D)
France	+ (ID-NAT)	0 (D)
Germany	0	0 (D/MP/ID-NAT†)
Greece	+ (ID-NAT/D/TSD)	+ (D)
Hungary	+ (D)	0 (D)
Ireland	0	+ (ID-NAT
Italy	+ (ID-NAT)	+ (D/ID-NAT†)
Latvia	0	0 (D)
Lithuania	0	0 (D)
Luxembourg	0	0 (D)
Malta	0	0 (D)
Netherlands	0	+ (D)
Poland	0	0 (D)
Portugal	+ (ID-NAT)	0 (D)
Romania	+ (2D-NAT†/D/TSD)	+ (D)
Slovakia	0	0 (D)
Slovenia	+ (ID-NAT‡)	0 (D)
Spain	+(ID-NAT)	0(D)
Sweden	0	+(D)
United Kingdom	0	+(MP-NAT)

*D, 28-d deferral of donors coming from affected areas; ID-NAT, individual donation NAT; 2D-NAT, 2 donations NAT; MP-NAT, minipool NAT; NAT, nucleic acid testing; TSD, temporary stop of donations in the area; +,  presence of cases; 0, absence of cases. More information about data collection in the different European Union countries is provided in the Appendix. †NAT screening in some blood banks. ‡NAT screening began in the end of summer 2018.

### ECDC’s WNV Maps

All blood banks in the EU use WNV maps to assess the travel-related risk of WNV infection among blood donors. Although the weekly updated maps are publicly available, some National Competent Authorities for Blood use ECDC WNV maps and supporting tables to produce national instructive documents for blood banks. Upon detection of a WNV NAT-reactive donor, blood banks retrieve and quarantine blood components derived from whole blood donated by the involved donor 120 days before the date of collection of the reactive donation and initiate a retrospective (lookback) analysis of recipients of potentially implicated blood components. There have been instances of observed differences between data on confirmed and probable human cases shown in the maps and reported by other sources (e.g., Rapid Alert System for Blood and Blood Components [RAB]), which is partially the result of occasional delays of longer than 10 days in reporting of cases to ECDC by some member states. These discrepancies could cause uncertainties for the map users and delays in the implementation of blood safety measures.

### WNV Infections among Blood Donors

During 2010–2017, blood banks in the affected areas of 7 EU countries (Austria, France, Greece, Italy, Portugal, Romania, and Spain) detected 152 WNV RNA–reactive donations among 2,636,653 donated blood units, corresponding to a mean frequency of 0.60 (range 0–2.95) positive donations/10,000 donations tested (Table 2). This estimation of WNV-positive donations in the EU is biased by the small amount of data from countries with sporadic outbreaks. In countries with established continuous WNV circulation in humans, such as Greece and Italy, the number of WNV-positive blood donations is proportional to the number of reported clinical cases in the general population, showing correlation coefficients of, for instance, R = 0.92 for Greece and R = 0.69 for Italy. Consequently, during the peak years, the positivity rate per 10,000 donations was 2.95 in Greece and 1.20 in Italy. In Austria, France, Spain, and Portugal, no positive blood donation was observed when only 1–3 autochthonous human WNV infections were detected in the population.

**Table 2:** Autochthonous and imported cases of WNV infection and WNV-positive blood donations by country in the European Union, 2009–2017*

Country and year	No. cases of WNV infection, autochthonous (imported)	No. WNV-positive blood donations	No. blood donations screened	WNV-positive blood donations/10,000 donations
Austria†				
2014	2	0	67,800	0.00
2015	6	5	74,677	0.67
2016	5	3	70,864	0.42
2017	6	2	67,544	0.30
France†				
2015	1	0	30,900	0.00
2017	2	0	4,044	0.00
Greece				
2010	262	8	27,108	2.95
2011	99 (1)	5	105,610	0.47
2012	160 (2)	4	36,911	1.08
2013	86	1	26,910	0.37
2014	15	0	6,662	0.00
2017	48	0	3,779	0.00
Italy				
2009	18	2	59,815	0.33
2010	11	6	118,295	0.51
2011	32	4	148,255	0.27
2012	73	14	116,255	1.20
2013	126 (3)	19	284,564	0.67
2014	24	4	334,356	0.12
2015	61(1)	16	322,196	0.50
2016	76 (5)	31	455,930	0.68
2017	57 (1)	25	338,900	0.74
Portugal				
2015	1	0	4,247	0.00
Romania‡				
2016	17	1	10,694	0.94
2017	16	1	11,390	0.88
Spain				
2010	2	0	10,768	0.00
2016	3	0	9,457	0.00

*USUV, Usutu virus; WNV, West Nile virus. †Austria corrected numbers of WNV positive cases and donations by excluding USUV-positive donations in 2016 and 2017. Other affected countries did not make corrections related to possible USUV positivity. ‡Data from affected areas where blood donations were screened.

### Transfusion-Transmitted WNV Infection

One report of WNV infectious blood donation noted that this donation resulted in TT-WNV infections in 2 patients (*31*). In 2012, the Greek Haemovigilance Centre reported these 2 TT-WNV infections: 1 patient received a platelet transfusion and developed WNND, and another patient received fresh frozen plasma and became positive for WNV but remained asymptomatic. Both blood components, including nontransfused erythrocytes, were prepared from a single whole blood donation and tested positive for WNV RNA after notification. These erythrocytes were discarded. The implicated donor, who retrospectively received a diagnosis of WNV infection, donated blood 8 days before a case of WNV infection in Greece was reported and preventive measures initiated (*31*).

### Threat to Blood Safety Posed by Emerging USUV

Because of NAT cross-reactivity, USUV-infected donations in the EU blood supply have been detected during routine screening of blood donations for WNV RNA. A USUV RNA–positive blood donor in the EU was detected in Germany in 2016 (*32*). In 2017, the follow-up investigation of 7 donors among 12,047 donations from eastern Austria whose blood tested positive by NAT (Cobas WNV assay; Roche Diagnostics, https://diagnostics.roche.com) showed by virus-specific NATs and sequencing that 6 of them had USUV infection, not WNV (*33*). Retrospective analyses of 4 blood donations among 70,864 donations from eastern Austria diagnosed as WNV-positive in 2016 showed 1 USUV-positive sample (*33*). In the 2018 transmission season, the highest-ever number of WNV and USUV NAT-positive blood donations were identified in eastern Austria (5 WNV NAT–positive, 17 USUV NAT–positive, and 1 WNV/USUV double infection among 31,598 blood donations) (*34*). Similarly, in the Lazio region of Italy, all 5 WNV NAT–reactive blood donations in 2017 and 2018 turned out to contain USUV, and not WNV, RNA (*35*). USUV antibodies in blood donors had been detected earlier. In 2009, four of 359 healthy blood donors were positive for USUV IgG in Italy (*36*), and in 2012, one of 4,200 screened blood donations tested positive for USUV IgG and IgM in southwest Germany (*37*).

## Discussion

The differences in the applied WNV blood safety measures in the EU countries with autochthonous human WNV transmission probably reflect the fact that the EU Blood Directives define measures only for donors returning from WNV-affected areas but not for donors residing in affected areas (*15*,*38*). Therefore, in the affected areas, each EU country applies blood safety interventions consistent with its national epidemiologic situation, economic capabilities, and experiences, as well as the EU preparedness plan for the prevention of TT-WNV (*17*), the European blood guide (*16*), recommendations from the US Food and Drug Administration (*39*), the American Association of Blood Banks standards (*40*), and other sources. Because of the smaller magnitude of outbreaks in affected EU countries, various blood safety measures are applied only locally. Conversely, driven by a continuous high number of WNV infection cases, an all-year universal donor screening has been implemented nationwide in the United States, but in Canada, donor screening is done only during the summer. Both countries switch from MP to ID-NAT depending on WNV activity levels (*39*,*41*). As predicted by models of environmental and climatic drivers (*42*), and after a substantial increase in human cases in 2018 (*43*), it is likely that WNV activity and the prevalence of infection in the donor population will increase across the EU in the future, which will inevitably call for greater screening of blood donations.

Changes in the epidemiology of existing infections and increasingly frequent emerging and reemerging infections may challenge the relevance of the current EU legislation on blood, tissues, and cells. Because the affected area at NUTS 3 level, which is currently used by EU countries in the donor selection procedure for travelers, could be considerably broader than the geographic area with ongoing transmission to humans, a new term should be developed for such affected areas to avoid misinterpretations. The role of ECDC in coordination of the development of common preparedness plans at the EU level (e.g., EU preparedness plans for the prevention of transfusion-transmitted WNV and Zika virus infections and safety of substances of human origin [*17*,*44*]) is to identify gaps in measures mandated through existing legislation and to achieve a high consistency and efficacy of implemented measures.

The mean frequency of 0.60 WNV RNA–positive donations/10,000 blood donations at the EU level is similar to data from the United States, where the American Red Cross detected 1,576 WNV-positive donations among 27 million donations during 2003–2012 (0.59 [range 0.18–1.49] positive/10,000 donations) (*45*). Observed data in the EU and United States show that the mean frequencies of WNV-positive blood donations in affected areas are low but not negligible. These data and experiences from the EU countries suggest that, in newly affected areas, initial cases of WNV transmission to humans tend to be sporadic, posing a low risk to potential blood donors, especially travelers. Such risk increases when more clinical cases are reported in the population, indicating that continuous transmission of WNV to humans has been established. However, in previously affected or endemic areas in Greece and Italy, WNV-positive donations had been reported before clinical cases were diagnosed in the general population (*46*–*48*). In Greece, lookback studies in 2010 of possible TT-WNV among patients with thalassemia revealed that some WNV-positive blood units might have been donated before the criteria used for initiating the implementation of blood screening with NAT were met (*48*). In Italy, circulation of the virus between birds and mosquitoes or a single case of transmission to a human or animal host are considered indicators of an increased risk of virus transmission to humans, and screening of blood donation starts even before the first case in humans is confirmed. Conversely, the absence of WNV-positive blood donated by travelers returning from affected areas calls for reevaluating triggers for implementation of traveler risk-related safety interventions because increased deferrals may have an effect on donor availability, especially during summer months.

Only 1 case of fully documented WNV transmission from a single donation to 2 recipients has been reported in the EU (*31*), which suggests that the current blood safety measures effectively prevent WNV infectious blood donations from entering the blood supply in the EU. However, besides blood safety interventions, other factors that constitute the risk of TT-WNV infection also contribute to the current situation. Considering that the projected incidence of WNV-positive donations generally correlates with WNND case frequencies (*49*), it is possible that the number of WNV-positive donations associated with the annual number of 30–356 cases of WNV infection in the EU (*30*) is too low for the occurrence of low-viremic donations that might escape safety interventions. In the United States, where substantially more WNV cases are reported annually, some breakthrough transfusion-transmitted cases occurred because of low-level viremia that escaped detection by MP-NAT (*50*). It is also believed that, despite the implemented surveillance and hemovigilance protocols and improvements in diagnostic tests, many WNV-like symptoms were undiagnosed, or the routes of infection were not investigated. Thus, a certain level of underrecognition and underreporting certainly contributes to the lack of TT-WNV cases. 

Recent molecular and serologic surveillance studies in Germany, Austria, and Italy identified USUV infections in blood donors (*32*,*33*,*36*,*37*). USUV is currently circulating more widely than WNV in the EU (*22*,*23*,*33*). Historically, USUV was introduced to Europe decades after WNV, although WNV activity was low in Europe before 2008. Because of the similar environmental requirements, mosquito vectors, and amplifying cycle in birds, USUV has easily been introduced in areas where WNV was present, resulting in a substantial geographic overlap in the circulation of these 2 viruses. USUV is currently spreading more intensively into new areas than WNV (*22*). Consequently, USUV but not WNV is currently circulating in the Netherlands, Belgium, and Switzerland (*33*). Furthermore, in Germany, only a few cases of WNV infections in birds were identified in 2018, whereas USUV is endemic throughout the country. No transfusion-associated USUV infection has been reported. However, the occurrence of USUV among blood donors is not fully determined because countries with USUV but without WNV circulation are not required to screen blood donations for flavivirus RNA. Assessing the risk of USUV transmission through blood transfusion and the clinical relevance of USUV infections in humans is therefore crucial. The currently used ID-NATs for blood screening are highly sensitive. The cross reactivity of these test systems with USUV (*33*,*34*) and potentially with other members of the Japanese encephalitis virus complex can contribute to the detection of these flaviviruses in donated blood. WNV NAT–reactive donations should therefore undergo virus-specific confirmatory tests to determine the actual flavivirus present in donated blood.

In summary, the paucity of reported TT- WNV cases provides reassurance about the efficacy of WNV blood safety interventions in the EU. However, the cocirculation of WNV and USUV in several EU countries, together with the yet unknown transfusion risk and clinical relevance of human USUV infections, needs further attention.

Appendix Additional information about West Nile virus and blood donations in European Union countries.
