# Supplementary material for: West Nile and Usutu Virus Infections and Challenges to Blood Safety in the European Union
Source: Emerg Infect Dis. 2019 Jun;25(6):1050–7. doi: 10.3201/eid2506.181755 (PMC6537739; doi:10.3201/eid2506.181755)
Supplement: Appendix — Additional information about West Nile virus and blood donations in European Union countries. [file 18-1755-Techapp-s1.pdf]

Article DOI: https://doi.org/10.3201/eid2506.181755

West Nile and Usutu Virus Infections and Challenges to Blood Safety in the European Union

Appendix

West Nile Virus and Blood Donations in Selected European Countries

Austria

Autochthonous human WNV infections in Austria were documented in 3 patients (2 in 2009 [1 WNND and 1 West Nile fever (WNF) case] and 1 WNND case in 2010) whose serum and cerebrospinal fluid samples tested positive during a retrospective examination in 2012 (*1*). Following countrywide measures based on the deferral of potentially exposed donors, WNV-NAT screening of blood donations was introduced in eastern Austria (federal states of Vienna, Lower Austria, and Burgenland) in 2014, and in the first year of screening 1 WNV NAT-positive blood donation was was identified (*2*,*3*). Seasonal screening of all donations between June 1 and November 30 was a measure to avoid deferrals of donors who had visited 1 of the 3 WNV-affected neighboring countries. Screening has been performed in pools of 19 samples, meeting the sensitivity limit enacted by Germany's Paul Ehrlich Institute of 250 copies WNV-lineage 2-RNA/mL (95% limit of detection) in the Roche Cobas 6800/8800 WNV assay. During 2014–2017, WNV nucleic acid was found in 10 out of 303,400 tested blood donations (*2*–*4*). In 2017, the follow-up of 7 donors from eastern Austria, whose blood reacted positive in the Cobas WNV assay among 12,047 donations, revealed that 6 of them had actually USUV and not WNV infection (*5*). In the other (not yet WNV affected) parts of Austria and offseason in eastern Austria, donors who have visited an affected country or region (based on the data of ECDC WNV maps at NUTS 3 level and other sources) are deferred for 28 days after having left the affected area. Although WNV positive donations have been detected, no transfusion-transmitted WNV infection was observed.

Croatia

Systematic WNV monitoring of animals started in 2012 and showed permanent seasonal circulation of the virus in Croatia. Monitoring is performed under the competence of the Ministry of Agriculture and in cooperation with the veterinary faculty in Zagreb (*6*). Clinical human cases of neuroinvasive WNV infection were detected in 2012 in eastern Slavonia. The seroprevalence of WNV infection in humans from that region in 2011 was 0.3% (*7*). During 2012–2017, the number of human WNV infections in Croatia varied by year (7 in 2012, 20 in 2013, 1 in 2014, 1 in 2015, 2 in 2016, 1 in 2017; data for 2017 not complete) and also occurred in the other parts of the country (*8*). From 2012 until March 16, 2018, there were no cases of WNV among blood donors in Croatia. Considering the possibility of blood transmission and potential hazards in patients after rapid alert sent by the Ministry of Health, the transfusion service in Croatia is undertaking the following measures: donors are deferred from donation for 28 days if they visited an affected area in the period from April 1 to November 30. ECDC maps showing human WNV cases are used to identify affected areas in the EU. Potential donors with confirmed or suspected WNV infections may donate blood 120 days after diagnosis. Postdonation information is reinforced. Cases of transfusion-transmitted WNV infection have not been reported.

France

The French Advisory Group for Substances of Human Origin is a multisector body responsible for providing recommendations on blood safety measures to be implemented in the case of arboviral epidemiologic alerts, which are managed according to French interministry guidelines. WNV blood safety measures were implemented for the first time during the equine outbreak of WNF in the Camargue in 2000. For local WNV transmission, the trigger for applying the measures is a confirmed case of WNV infection in humans. Once implemented, blood safety measures last until the end of the mosquito season. The measures in the affected area comprise the cancellation of blood collection sessions or temporary deferral of donors for 28 days unless WNV NAT screening and/or pathogen reduction method for platelets and plasma are implemented. Potential blood donors visiting affected areas in France or abroad are temporarily deferred for 28 days after return. When applied, blood donation screening uses ID-NAT testing (Procleix WNV assay on the Grifols Tigris System, https://www.grifols.com). In 2015, 1 human WNF case in the department Gard, and 41 equine WNF cases in departments 13, 30, and 34 were detected. During this outbreak, 30,900 blood donations were screened using ID-NAT during

September–November 2015; all were negative for WNV RNA. During October 13–November 27, 2017, 1 equine and 2 human WNF cases in the department of Alpes Maritimes were reported. Retrospective testing of cerebrospinal fluid and/or serum samples from 61 neurologic patients in August and September at the Hospital of Nice were all WNV negative. All donations tested in ID-NAT WNV (n = 4044) by the French National Blood Service during October 20–November 21 were negative. By the time of the expert meeting, no donors were found positive and no cases of transfusion-transmitted WNV infection were diagnosed in France.

Germany

Monitoring of migratory and resident birds for zoonotic arbovirus infections showed that WNV was not present in Germany before March 2018. Although WNV-specific RNA was not detected, USUV was already widespread in birds in Germany. In addition, neutralizing antibodies to WNV and USUV were identified (*9*,*10*). Human arbovirus infections, which were insufficiently reported in Germany under "other threatening diseases," became an explicit reporting obligation from May 2016 onward. Overall, 10 imported cases of WNV infections were reported in Germany. WNV substances of human origin safety measures entail donor deferral for 4 weeks after residence in an area with ongoing transmission of WNV to humans for >2 consecutive days, starting after leaving this area. If the donor/donation WNV-NAT testing is applied, the minimum sensitivity of 250 copies of WNV-RNA/mL related to a single donation and concurrent detection of WNV lineages 1 and 2 are required. Affected areas are countries considered with $\geq$1 confirmed autochthonous human WNV cases in the current and the previous year. Areas for geographic deferral of visitors are defined as regions at NUTS 3 level (Eurostat definition). For the donor selection procedure, blood banks use the list of affected areas in the EU, which is based on the ECDC Surveillance Atlas and published on the homepage of the Paul Ehrlich Institute (www.pei.de/wnv-spenderrueckstellung). Updates are performed on the first working day of each month. Data from the ECDC Surveillance Atlas are exported and transformed to meet only confirmed cases and translated into German. Measures are applied from June 1 to November 30.

Greece

Surveys, undertaken in some parts of Greece before 2010, showed that ≈1% of the population had WNV antibodies, indicating a circulation of WNV or related viruses in the observed areas, but no clinical disease or WNV NAT positivity in humans were detected (*11*,*12*).

In 2010, the first outbreak of WNV infection in Grece was recorded as the largest in Europe since 1996 (*13*). WNV lineage 2–Nea Santa was introduced into Greece in or before 2010 and has since been responsible for 672 diagnosed symptomatic clinical cases, of which 30% were WNF and 70% were WNND; 77 patients with WNND died (*13*,*14*). Raising awareness among physicians and susceptible populations (the elderly and persons with concurrent conditions) and enhanced surveillance during mosquito transmission season were implemented. The occurrence of human cases in 5 consecutive years (2010–2014) and in 2017 suggests that WNV lineage 2 has become established in Greece (*13*,*15*). A study of symptomatic WNV lineage 2 in Greece suggests blood group A RhD negativity as a new genetic risk factor associated with WNV infection and level of illness. The possibility that HLA C*08, DRB1*O4:O5, and DQB1*O2 are protective alleles and DRB1*10:O1 a "susceptible" allele to WNV infection and the role of secretor status of an individual (defined by the phenotype in the Lewis blood group) in relation to this infection has also been suggested (*16*). Blood safety and hemovigilance measures are in conformity with Directives 2004/33/EC, 2014/110/EU (*17*,*18*). A trigger for the implementation of measures would be the first laboratory confirmed human case of WNV infection. In addition to donation screening in affected areas defined at NUTS3 level, the quarantine of blood components collected 15 days before starting the measures, enhanced postdonation and posttransfusion information have also been in place since 2010 (*15*). ECDC WNV maps are in use for the selection of donors traveling abroad. Additional information on affected areas is obtained from the Rapid Alert System for Blood and Blood Components (RAB) platform. During 2010–2017 the annual number of affected NUTS 3 areas with human cases varied from 4 (2014) to 19 (2013), with only equine cases from 3–8 areas, and with both human and equine cases from 2–5 areas. In the same period, the rate of reactive donations for WNV-RNA was 0.87/10,000 (from 206,980 donations tested by ID-NAT, 18 were positive, for a frequency of 1:11,498 donations). Before the start of NAT screening of blood donations in 2012, WNV infection was transmitted to 2 patients who received transfusions of 2 different blood components (fresh frozen plasma and platelets) derived from the same blood unit of an asymptomatic blood donor. One of the 2 infected patients, who received a transfusion of whole blood derived platelets, had severe transfusion-transmitted WNND (*13*).

Hungary

Hungary was the first country in Europe in which the lineage 2 WNV emerged (*19*). During 2003–2007, a yearly average of 6 cases of WNND were diagnosed (*19*). In 2010, a lineage 2 WNV outbreak in humans, including several cases of encephalitis, was observed. Public health measures to prevent WNV transmission are in place from April to the end of November annually and include enhanced human and animal surveillance, as well as vector control measures. Blood safety measures are based on blood donor deferral for 28 days after being in an affected area for >24 hours. Measures in the country are valid until the end of the mosquito season. During 2010–2017, Hungary reported to ECDC 163 probable and confirmed cases of WNV infection in the population. Data on positive blood donations/donors are not available. Cases of transfusion-transmitted WNV infection have not been reported in Hungary.

Italy

Following the first human cases of WNV infection identified in the Emilia-Romagna region in 2008, Italy implemented a 28-day deferral policy of blood donors living in areas with ongoing WNV transmission. During June–November 2009–2014, the National Blood Centre used WNV NAT screening of blood donations in provinces corresponding to NUTS 3 units where the virus had been circulating among animals and mosquitoes or where a human case of WNND and/or a WNV NAT-positive donor had been confirmed. Besides NAT screening, the National Blood Centre enforced a nationwide 28-day deferral for blood donors who had spent ≥1 night in areas with active WNV circulation. In 2008, an integrated surveillance targeting mosquitoes, birds, and humans was put into effect starting in the Emilia-Romagna region; in the following years, this surveillance was extended to 4 other regions (Piedmont, Lombardy, Friuli Venetia Giulia, and Veneto) (*20*).The remaining regions adopted the national surveillance plan. Currently, the triggers for WNV ID-NAT screening of blood donations in the June–November period are notification of WNV circulation in mosquitoes and/or wild birds in the regions where the integrated surveillance is in place, notification of a WND equid case, and/or notification of WNV infection in humans. WNV NAT screening continues until the end of the surveillance season in November (*20*). Nationwide, all donors having spent ≥1 night in the Italian provinces where NAT testing has been introduced are deferred for 28 days unless tested negative by ID-NAT. The same protocol is adopted for donors coming from the United States, Canada, and any other EU or non-EU countries where WNV human cases were reported by the ECDC. Other

general measures include strengthening of the predonation questionnaire and donor physical examination, enforcing postdonation information, implementation of ad hoc hemovigilance procedures, and communication and collaboration among all institutional healthcare bodies involved. The number of screened donations was ≈150,000 in 2011 and ≈340,000 in 2017, with a peak in 2016 (≈460,000 tested donations). The number of WNV-positive donations also increased, from 4 in 2011 to 25 in 2017, with a peak of 31 positive donations in 2016. In 2017, 27 cases of WNND were confirmed in Italy. Specific measures for hematopoietic stem cell donations (WNV NAT testing and 28-day deferral criteria, if applicable, on the basis of transplant timing) were applied in Emilia-Romagna, Veneto, Lombardy, Piedmont, and Sardinia. There are no records of transfusion-transmitted WNV infection in Italy.

Portugal

The National Hemovigilance Office, which is responsible for blood safety monitoring in Portugal, obtains data from the weekly bulletin issued by the Directorate General of Health, the European Commission platform RAB, and ECDC's WNV maps. In Portugal, only 4 human autochthonous WNV cases have been reported: 3 cases in the Algarve and 1 in the Sado Valley of the Lisbon NUTS 3 region. Three of these cases were classified as probable cases: 2 cases detected in 2004 (*21*) and 1 case in 2010. The first confirmed case was in 2015 (Algarve). Following this case and in line with the Blood Directives, the blood safety measures have been implemented during August 31–November 4, 2015 (*22*). In the affected area, blood collection is halted until the implementation of WNV ID-NAT screening, which lasts until the end of the mosquito season; blood components in stock are quarantined and retrospectively screened by WNV ID-NAT testing, when possible. If available, pathogen inactivation of plasma and platelets is implemented. Potential blood donors who spent ≥1 night in an affected area are deferred for a period of 28 days unless tested negative by ID-NAT. Blood services defer confirmed cases of WNV infection for 120 days after recovery and enhance postdonation information and posttransfusion hemovigilance. During the outbreak in 2015, 4,274 blood donations were screened during September 4–November 4; no WNV RNA-positive blood donation was found. There were no reports of transfusion-transmitted WNV infection or decreases in the number of blood donations in the affected area. The number of potential blood donors deferred in nonaffected areas could not be assessed. During 2015–2017, 108 equine WND cases were identified in the Algarve, Alentejo, and Sado Valley.

Romania

Circulation of WNV in Romania has been reported since the 1950s. Following the largest outbreak of human WNV infection in Europe to date in 1996, when ≈400 clinical cases of WNND disease were reported in Bucharest (*23*), Romania implemented a WNV surveillance system. During 1997–2009, sporadic human cases were reported in the southern part of the country. In 2010, WNV reemerged, with 52 human cases in the central and northern part of the country. Blood safety measures have been implemented since 2010 by the National Center for Prevention and Control of Infectious Diseases, which also elaborates on and disseminates the methodology for the surveillance and control of WNV infection, assesses the risk of transfusion-transmitted WNV infection, and informs all stakeholders, including the veterinary agency, about human WNV cases. Blood safety measures are in line with the current EU directives and defined in the preparedness plan. Based on information provided by ECDC and RAB, blood banks apply a 28-day deferral for potential donors who have previously traveled to affected areas inside or outside the country. Specific measures in affected villages and small towns include temporary deferral for resident donors, suspension of mobile blood collection sessions during the season, and 28 days deferral for potential donors having traveled in affected areas. Based on the risk assessment in larger towns, ID-NAT or a pool of 2D-NAT is implemented or there is a temporary deferral of resident donors, 14-day quarantine of donated blood, and release after post-donation information. It is estimated that ≈1,000 prospective donors are temporarily deferred nationwide each year. Hospitals in affected areas are strengthening the rational use of blood and perform the lookback procedure if blood from a patient who has received a transfusion is found positive for WNV. Cases of transfusion-transmitted WNV infection have not been reported. During 2010–2017, the estimated risk of viremic donations from asymptomatic donors among the released units varied from 1.3 to 13 per 100,000 blood units. WNV ID and pool 2D-NAT testing during August 17–October 31, 2016 showed 1 positive per 10,694 tested donations (0.93/10,000). In 2017 (2D pool NAT), there was 1 positive donation in 11,390 donations (0.88/10,000).

Spain

The circulation of WNV in birds in Spain was documented in 2004 when, according to retrospective analysis, the first human case of WNV infection occurred (*24–26*). Two confirmed human cases in Cádiz and 43 equine cases in Cádiz, Huelva, Malaga, and Sevilla were reported

in September 2010. Since 2010, the System for Epidemiologic Vigilance in Andalusia runs an active WNV surveillance in humans from April to the end of November each year. During 2011–2015, the virus was detected in horses and wild birds. Three WNV human cases were diagnosed in 2016. The trigger for the implementation of blood safety measures is a confirmed locally acquired WNV infection in Spain. The Spanish Society of Blood Transfusion provides a list of the Spanish group of diseases transmissible by transfusion to all blood collection centers with weekly updates and a web link to the ECDC maps. Preventive measures include the deferral for 28 days of donors who have spent ≥1 night in an affected area and deferral for 120 days for donors with a diagnosis of WNF or who present or have presented a clinical picture compatible with WNV infection. In areas of 10 km around the central focus of a locally transmitted case, blood banks cancel blood collections and quarantine or retest by ID-NAT the blood components in stock that are derived from blood donated 15 days before the first case of WNV infection was reported. The recovered and plasmapheresis plasma from affected areas can be used for fractionation. Donors are informed to report to the blood bank whether they developed symptoms of WNF within 14 days after the donation. There was no WNV RNA-positive result among 10,768 screened in 2010 and 9,457 blood donations screened in 2016.

United Kingdom

The primary strategy for minimizing the risk of WNV infection in the UK population is surveillance. Activities targeting humans, animals, and vector sources have been in place since 2002; these include passive surveillance of equids and wild birds and targeted surveillance of mosquitoes. This program has identified the *Culex modestus* mosquito, the bridge WNV vector in continental Europe, in the 2 counties in the river Thames estuary, where the vector has been endemic since 2010. According to the WNV risk assessment published by the Human Animal Infections and Risk Surveillance group in December 2017 (*27*), there continues to be no evidence of WNV presence in the UK, with only 3 travel-related cases having been reported: 2 in 2014 (from Egypt and the United States) and 1 in 2017 (from South Africa). The Human Animal Infections and Risk Surveillance group recommended continued monitoring of vector and host populations, and surveillance and promotion of awareness among clinicians to encourage appropriate testing of clinical cases in the UK. To protect safety and sufficiency of the blood supply, WNV ID-NAT screening was implemented in 2012, replacing the 28-day deferral of donors who have visited areas with ongoing WNV activity. The Donor Selection Guidelines

devised by the Joint United Kingdom Blood Transfusion and Tissue Transplantation Services Professional Advisory Committee stipulates deferral of 6 months after proven WNV infection or development of compatible symptoms within 28 days after return from an affected area and 28 days after return where there has been no diagnosis nor symptoms. A negative result using a validated WNV NAT allows donor acceptance in the asymptomatic group. This discretionary screening is applied each year during the May–November mosquito season, using mini-pools of 6 donations. During 2012–2017, the National Health Services Blood and Transplant tested 185,258 donations, accounting for ≈2% of all blood donations. There have not been any confirmed positive WNV RNA results in this period. If countries that receive high numbers of visitors from the UK (e.g., Spain, France, Portugal), were to become affected WNV areas, testing in England would increase, thereby influencing the cost-effectiveness of screening versus deferral.

West Nile Virus and Blood Donations in Other EU Countries

In 2018, the European Blood Alliance (EBA) performed a questionnaire survey of the WNV blood safety measures applied by EBA members. Among 14 EBA members from the EU, WNV circulation and transmission was absent in 10 countries (Belgium, Denmark, Finland, Germany, Ireland, Malta, the Netherlands, Sweden, Switzerland, United Kingdom), whereas in Austria, France, Slovenia, and Spain, transmission occurred sporadically. EBA has a global partnership with the Alliance of Blood Operators (Canadian Blood Service, America's Blood Centers, American Red Cross, the Australian Red Cross Blood Service, Blood Systems Inc., and the NHS Blood and Transplant). In the survey, Australia reported sporadic WNV cases, whereas the United States was endemic. In the EU countries, surveillance systems are in place, although the monitoring is mostly passive and triggered by animal and/or human cases. ECDC maps displaying human WNV cases are helpful and appropriate for identifying WNV-affected areas for the selection or exclusion of donors. Discrepancies between the number of reported cases by ECDC and other sources have been observed. Confirmatory testing takes time, which prolongs reporting. For the purpose of applying blood safety measures, only confirmed cases are relevant; however, some EU countries have no capacity to perform confirmatory tests. Reporting of additional information about cases has been suggested and a tighter timeline for reporting emphasized.

Most EU countries apply a donor deferral of 28 days after leaving a WNV-affected area. In the UK and in Ireland, where traveling donors are screened by WNV NAT, and in France, where donors in affected areas have been tested, no WNV RNA-positive donations were found. In the WNV-endemic area of eastern Austria, several WNV RNA positive blood donors have been identified; however, USUV RNA has been found more frequently since 2017 (*5*). Estimation performed by the European Up-Front Risk Assessment Tool shows a very low risk for blood safety posed by WNV, especially for blood banks in nonaffected countries. The EBA emerging infectious diseases monitoring group concluded that the current measures to prevent transfusion-transmitted WNV infection in nonaffected countries tend to be disproportional if the risk remains very low. The group also emphasized that there is a need to discuss the appropriateness of triggers for implementing a geographic deferral or screening of travelers to affected areas.

References

1. Aberle S, Heinz FX. Virus epidemiologic information No. 12/12. West Nile virus infections in Austria. 2012 [cited 2018 Mar 11]. http://www.virologie.meduniwien.ac.at/fileadmin/virologie/files/Epidemiologie/2012/1212s.pdf

2. Jungbauer C, Hourfar MK, Stiasny K, Aberle SW, Cadar D, Schmidt-Chanasit J, et al. West Nile virus lineage 2 infection in a blood donor from Vienna, Austria, August 2014. J Clin Virol. 2015;64:16–9. http://dx.doi.org/10.1016/j.jcv.2015.01.003

3. Kolodziejek J, Seidel B, Jungbauer C, Dimmel K, Kolodziejek M, Rudolf I, et al. West Nile virus positive blood donation and subsequent entomological investigation, Austria, 2014. PLoS One. 2015;10:e0126381. http://dx.doi.org/10.1371/journal.pone.0126381

4. Kolodziejek J, Jungbauer C, Aberle SW, Allerberger F, Bagó Z, Camp JV, et al. Integrated analysis of human-animal-vector surveillance: West Nile virus infections in Austria, 2015–2016. Emerg Microbes Infect. 2018;7:1–15. http://dx.doi.org/10.1038/s41426-018-0021-5

5. Bakonyi T, Jungbauer C, Aberle SW, Kolodziejek J, Dimmel K, Stiasny K, et al. Usutu virus infections among blood donors, Austria, July and August 2017—raising awareness for diagnostic challenges. Euro Surveill. 2017;22. http://dx.doi.org/10.2807/1560-7917.ES.2017.22.41.17-00644

6. Barbić L, Vilibić-Čavlek T, Stevanović V, Savić V, Klobučar A, Pem-Novosel I, et al. [“One health”—detection and surveillance of emerging and re-emerging arboviruses in Croatia]. Croat J Infect. 2015;35:53–60.

7. Vilibić-Cavlek T, Barbić L, Ljubin-Sternak S, Pem-Novosel I, Stevanović V, Gjenero-Margan I, et al. [West nile virus infection: re-emergent disease in Croatia]. Lijec Vjesn. 2013;135:156–61.

8. Health CIoP. Communicable diseases in Croatia in 2016 [cited 2018 Feb 15]. https://www.hzjz.hr/en/news/communicable-diseases-in-croatia-in-2016

9. Michel F, Fischer D, Eiden M, Fast C, Reuschel M, Müller K, et al. West Nile virus and Usutu virus monitoring of wild birds in Germany. Int J Environ Res Public Health. 2018;15:171. http://dx.doi.org/10.3390/ijerph15010171

10. Ziegler U, Seidowski D, Angenvoort J, Eiden M, Müller K, Nowotny N, et al. Monitoring of West Nile virus infections in Germany. Zoonoses Public Health. 2012;59(Suppl 2):95–101. http://dx.doi.org/10.1111/zph.12015

11. Papa A, Perperidou P, Tzouli A, Castilletti C. West Nile virus—neutralizing antibodies in humans in Greece. Vector Borne Zoonotic Dis. 2010;10:655–8. http://dx.doi.org/10.1089/vbz.2010.0042

12. Kantzanou MN, Moschidis ZM, Kremastinou G, Levidiotou S, Karafoulidou A, Politis C, et al. Searching for West Nile virus (WNV) in Greece. Transfus Med. 2010;20:113–7. http://dx.doi.org/10.1111/j.1365-3148.2009.00964.x

13. Pervanidou D, Detsis M, Danis K, Mellou K, Papanikolaou E, Terzaki I, et al. West Nile virus outbreak in humans, Greece, 2012: third consecutive year of local transmission. Euro Surveill. 2014;19:20758. http://dx.doi.org/10.2807/1560-7917.ES2014.19.13.20758

14. Papa A, Bakonyi T, Xanthopoulou K, Vazquez A, Tenorio A, Nowotny N. Genetic characterization of West Nile virus lineage 2, Greece, 2010. Emerg Infect Dis. 2011;17:920–2. http://dx.doi.org/10.3201/eid1705.101759

15. Politis C, Tsoukala A, Hatzitaki M, Pappa A, Englezou A, Zafeiriou C, et al. West Nile virus (WNV) outbreak in Greece and blood safety measures. Vox Sang. 2011;101(Suppl1):42.

16. Politis C, Parara M, Kremastinou J, Hasapopoulou E, Iniotaki A, Siorenta A, et al. Associations of ABO, D, and Lewis blood groups and HLA class I and class II alleles with West Nile virus lineage 2 disease outcome in Greece, 2010 to 2013. Transfusion. 2016;56:2115–21. http://dx.doi.org/10.1111/trf.13667

17. European Commission. Commission Directive 2004/33/EC of 22 March 2004 implementing Directive 2002/98/EC of the European Parliament and of the Council as regards certain technical requirements for blood and blood components, 2004 [cited 2018 Apr 25]. http://eur-lex.europa.eu/LexUriServ/LexUriServ.do?uri=OJ:L:2004:091:0025:0039:EN:PDF

18. European Commission. Commission Directive 2014/110/EU of 17 December 2014 amending Directive 2004/33/EC as regards temporary deferral criteria for donors of allogeneic blood donations 2014 [cited 2018 Apr 25]. http://eur-lex.europa.eu/legal-content/EN/TXT/?uri=OJ%3AJOL_2014_366_R_0011

19. Krisztalovics K, Ferenczi E, Molnar Z, Csohan A, Ban E, Zoldi V, et al. West Nile virus infections in Hungary, August–September 2008. Euro Surveill. 2008;13:19030.

20. Velati C, Angelini P, Pupella S. State of the art: West Nile virus circulation surveillance in Italy and transfusion risk early prevention methods. Transfus Clin Biol. 2017;24:172–5. http://dx.doi.org/10.1016/j.tracli.2017.06.019

21. Connell J, Garvey P, Cotter S, Conway A, O'Flanagan D, O'Herlihy BP, et al. Two linked cases of West Nile virus (WNV) acquired by Irish tourists in the Algarve, Portugal. Euro Surveill. 2004;8:2517. https://dx.doi.org/10.2807/esw.08.32.02517-en

22. Lopez LA, Yang SJ, Hauser H, Exline CM, Haworth KG, Oldenburg J, et al. Ebola virus glycoprotein counteracts BST-2/Tetherin restriction in a sequence-independent manner that does not require tetherin surface removal. J Virol. 2010;84:7243–55. http://dx.doi.org/10.1128/JVI.02636-09

23. Tsai TF, Popovici F, Cernercu C, Campbell GL, Nedelcu NI. West Nile encephalitis epidemic in southeastern Romania. Lancet. 1998;352:767–71.

24. Figuerola J, Jiménez-Clavero MA, López G, Rubio C, Soriguer R, Gómez-Tejedor C, et al. Size matters: West Nile Virus neutralizing antibodies in resident and migratory birds in Spain. Vet Microbiol. 2008;132:39–46. http://dx.doi.org/10.1016/j.vetmic.2008.04.023

25. López G, Jiménez-Clavero MA, Tejedor CG, Soriguer R, Figuerola J. Prevalence of West Nile virus neutralizing antibodies in Spain is related to the behavior of migratory birds. Vector Borne Zoonotic Dis. 2008;8:615–21. http://dx.doi.org/10.1089/vbz.2007.0200

26. Bernabeu-Wittel M, Ruiz-Pérez M, del Toro MD, Aznar J, Muniain A, de Ory F, et al. West Nile virus past infections in the general population of southern Spain. Enferm Infecc Microbiol Clin. 2007;25:561–5. http://dx.doi.org/10.1157/13111181

27. Human Animal Infections and Risk Surveillance (HAIRS) group. Qualitative assessment of the risk that West Nile virus presents to the UK human population 2017 [cited 2018 Mar 11]. https://www.gov.uk/government/publications/hairs-risk-assessment-west-nile-virus
